# Distinct Roles of Wnt/*β*-Catenin Signaling in the Pathogenesis of Chronic Obstructive Pulmonary Disease and Idiopathic Pulmonary Fibrosis

**DOI:** 10.1155/2017/3520581

**Published:** 2017-05-09

**Authors:** Juan Shi, Feng Li, Meihui Luo, Jun Wei, Xiaoming Liu

**Affiliations:** ^1^Center of Laboratory Medicine, General Hospital of Ningxia Medical University, Yinchuan 750004, China; ^2^Institute of Human Stem Cell Research, General Hospital of Ningxia Medical University, Yinchuan, Ningxia 750004, China

## Abstract

Wnt signaling pathways are tightly controlled under a physiological condition, under which they play key roles in many biological functions, including cell fate specification and tissue regeneration. Increasing lines of evidence recently demonstrated that a dysregulated activation of Wnt signaling, particularly the Wnt/*β*-catenin signaling, was involved in the pathogenesis of chronic pulmonary diseases, such as chronic obstructive pulmonary disease (COPD) and idiopathic pulmonary fibrosis (IPF). In this respect, Wnt signaling interacts with other cellular signaling pathways to regulate the initiation and pathogenic procedures of airway inflammation and remodeling, pulmonary myofibroblast proliferation, epithelial-to-mesenchymal transition (EMT), and development of emphysema. Intriguingly, Wnt/*β*-catenin signaling is activated in IPF; an inhibition of this signaling leads to an alleviation of pulmonary inflammation and fibrosis in experimental models. Conversely, Wnt/*β*-catenin signaling is inactivated in COPD tissues, and its reactivation results in an amelioration of airspace enlargement with a restored alveolar epithelial structure and function in emphysema models. These studies thus imply distinct mechanisms of Wnt/*β*-catenin signaling in the pathogenesis of these two chronic pulmonary diseases, indicating potential targets for COPD and IPF treatments. This review article aims to summarize the involvement and pathogenic roles of Wnt signaling pathways in the COPD and IPF, with a focus on the implication of Wnt/*β*-catenin signaling as underlying mechanisms and therapeutic targets in these two incurable diseases.

## 1. Introduction

Chronic obstructive pulmonary disease (COPD) and idiopathic pulmonary fibrosis (IPF) are two severe, chronic pulmonary diseases with distinct clinical and pathological features. Clinicopathologically, COPD is characterized by a progressive and poorly reversible airflow limitation caused by a concurrence of airway inflammations and emphysema, while IPF features an impaired diffusion capacity with a restrictive pattern of lung volume abnormality [1, 2]. While therapeutic interventions are available for ameliorating manifestations of COPD and IPF, there is no curative option currently available, partially owing to the largely unknown pathogenesis of these devastating disorders. Mechanistically, many signaling pathways are involved in the pathogenesis of COPD and IPF. Among them, the transforming growth factor beta (TGF-*β*) signaling is one of the most studied pathways. Of great interest, developmental signaling pathways, such as wingless-type MMTV integration site (Wnt) pathways, have recently gained increased attention in the onset and progression of chronic pulmonary diseases, particularly in asthma, COPD, and IPF. In this respect, dysregulated Wnt signaling has been implicated in lung remodeling, pulmonary myofibroblast proliferation, epithelial-to-mesenchymal transition (EMT), and development of emphysema [3].

The Wnt pathways are developmental signaling pathways that play pivotal roles in cell fate specification, cell migration and polarity, organogenesis, stem cell self-renewal, and tissue homeostasis under a physiological condition and tissue repair following injuries [4]. With respect to the lung, the expression of various Wnt ligands, including Wnt2, Wnt3A, Wnt5A, Wnt5B, Wnt7B, Wnt 10A, Wnt11, and Wnt13, has been detected in both developing and adult lung [5]. In developing lung, Wnt signaling exerts functions in branching morphogenesis and airway formation [6, 7]. In mature lungs, Wnt signaling is often reactivated during injury repair and tissue regeneration. Moreover, accumulating evidence has demonstrated that dysregulated Wnt signaling is a key contributor to pathogenesis of hyperproliferative lung diseases such as COPD and IPF [3, 7–9]. Clinically, activation of Wnt/*β*-catenin signaling, accompanied with an increased expression of its target genes (i.e., Cyclin D1 and MMP-7), was observed in airways of patients with IPF [10, 11]. In patients with emphysema, repressed Wnt/*β*-catenin signaling activity and reduced expression of its target genes were reported [12, 13]. Interestingly, a reactivation of this signaling led an attenuation of experimental emphysema in vivo [14]. The distinct roles of Wnt/*β*-catenin in the onset and progression between IPF and COPD suggest the complicity of roles of this signaling in chronic pulmonary diseases. An in-depth understanding of the mechanisms underpinning Wnt signaling-regulated pathogenesis is therefore important for prevention and management of these incurable diseases. In this review article, we attempt to scrutinize recent findings in pathogenic roles of Wnt signaling in COPD and IPF, with a main focus on molecular mechanisms by which the Wnt signaling regulates pathogenesis of these disorders and the potential of this signaling as therapeutic targets for disease treatments.

## 2. Wnt Signaling Pathways

To date, 19 gene encoding distinct Wnt protein ligands have been identified in humans [15]. In the presence of Wnt ligand, the Wnt protein first binds to a seven-transmembrane-spanning frizzled (FZD) receptor that may also complex with coreceptors such as the low-density lipoprotein-related receptor (LRP) 5 or 6 [15]. Based on the dependence of Wnt signaling effector, *β*-catenin, Wnt signaling pathways can be further characterized by a “canonical pathway” and several “noncanonical pathways.” The canonical Wnt pathway is also known as the “Wnt/*β*-catenin pathway,” and the noncanonical pathways include the planer cell polarity (PCP), c-Jun N-terminal protein kinases (JNK), protein kinase C/calcium (PCK/Ca^2+^), receptor-like tyrosine kinase (RYK), and receptor tyrosine kinase-like orphan receptor (Ror) pathways [4, 15, 16]. Among these, the Wnt/*β*-catenin signaling pathway is the most investigated and best characterized.

**Figure 1:**
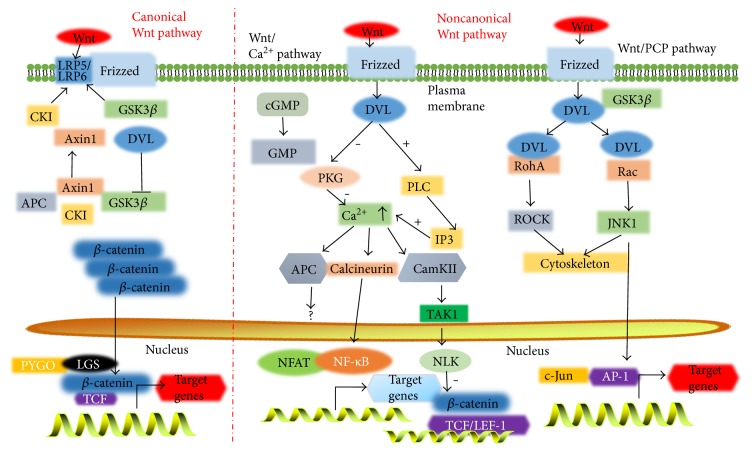
Illustration of Wnt signaling cascades. Based on the dependence of *β*-catenin, Wnt signaling can be classified as *β*-catenin-dependent Wnt signaling pathway (canonical Wnt signaling pathway) and *β*-catenin-independent Wnt signaling pathway (noncanonical Wnt signaling pathway). In canonical Wnt signaling pathway, in the absence of Wnt ligand(s), cytoplasmic *β*-catenin is targeted for phosphorylation by a multiprotein complex comprised of Axin, adenomatous polyposis coli (APC), glycogen synthase kinase 3*β* (GSK3*β*), and casein kinase 1*α* (CK1*α*). The phosphorylated form of *β*-catenin is recognized by an E3 ubiquitin ligase (*β*-TrCP) and then targeted for proteosomal degradation, resulting in low cytosolic levels (left panel); in the presence of Wnt ligand(s), Wnt ligand binds to a FZD and LRP coreceptors, which triggers a signaling cascade by activating Dvl. The activated Dvl inhibits GSK3*β*, which destroys the stability of the multiprotein complex and accordingly leads to the intracellular accumulation of cytosolic *β*-catenin. The stable and active *β*-catenin then translocates into the nucleus, in which it acts as a transcriptional coactivator with TCF/LEF to activate Wnt-responsive target genes (left panel). In a noncanonical Wnt signaling pathway, Wnt ligand (such as the Wnt5a, a typical noncanonical Wnt) binds to its receptor (FZD) and coreceptor (Ror1/2) and triggers noncanonical signaling cascades, which include the Wnt/calcium (Ca^2+^) and Wnt/planar cell polarity (PCP) pathways. In the Wnt/Ca^2+^ pathway (left panel), Wnt protein binds to FZD and Ror2 receptor and leads to activated G proteins, resulting in enhanced intracellular calcium level or decreased cGMP; the calcium/calmodulin-dependent protein kinase II (CaMKII) or protein kinase C (PKC) was then activated. In the Wnt/PCP pathway (right panel), the Wnt ligand binds to a FZD receptor on the cell surface, followed by activating Rho/Rac small GTPase and Jun N-terminal kinase (JNK) to assist with cytoskeletal organization and gene expression (right panel).

The Wnt/*β*-catenin pathway is characterized by Wnt binding to its coreceptor complex (that constituted by the LRP5 or LRP6) and to a member of the ten FZD family of proteins (Figure 1()) [17, 18]. In a steady state without a Wnt ligand, the cytosolic *β*-catenin is phosphorylated by a complex consisting of glycogen synthase kinase 3*β* (GSK3*β*), casein kinase I (CKI), Axin, and adenomatous polyposis coli (APC) [19]. In this context, Axin is a scaffold that favors the formation of a complex with GSK3*β* and APC. Once in the complex, the GSK3*β* promotes cytosolic *β*-catenin phosphorylation and APC mediates phosphorylated *β*-catenin binding to the ubiquitin-mediated proteolysis pathway in cytoplasm. In the presence of a Wnt protein ligand, Wnt binds to its coreceptor complex and activates Wnt signaling by recruiting the cytosolic disheveled (Dvl) proteins and blocking or destroying Axin/GSK3*β*/APC complex formation, which in turn inhibits *β*-catenin degradation and thus leads to an accumulation of *β*-catenin in the cytoplasm. The accumulated cytosolic *β*-catenin is then translocated into the nucleus and bind to transcriptional factors T-cell factor/lymphoid enhancer factor 1 (TCF/LEF1), which initiates the expression of Wnt target genes [15].

A Wnt signaling pathway that is independent of *β*-catenin-TCF/LEF1 is classified into a “noncanonical signaling pathway” (Figure 1), which may regulate both transcriptional and nontranscriptional responses in cells [20]. The planar cell polarity (PCP) and the Wnt/Ca^2+^ pathways are two of the best-characterized *β*-catenin-independent Wnt pathways [21]. The Wnt/PCP pathway is characterized by a Dvl-driven sorting of cellular components to either the proximal or distal region of cells and directs it within the tissue [22]. Upon binding to FZD receptor, Wnt activates a cascade of the small GTPases RAC1 and Ras homolog gene family member A (RHOA) and JNK exerts downstream effectors that asymmetrically direct cytoskeletal organization and coordinate polarization of cells within the plane of epithelial sheets by controlling target gene expression [23]. In the Wnt/PKC/Ca^2+^ pathway, a Wnt ligand binding to an FZD receptor triggers the activation of heterotrimeric G proteins, which are in turn able to activate phospholipase C (PLC) and PKC, thus leading to the release of intracellular Ca^2+^. The evoked Ca^2+^ concentration activates the phosphatase calcineurin, which leads to dephosphorylation of transcription factor nuclear factor of activated T-cells (NFAT) to regulate the transcription of genes controlling cell fate and cell migration [21].

Interestingly, Wnt signaling can also be regulated both by intracellular proteins which influence signal transduction and by extracellular antagonists such as Wise (Sostdc1), secreted frizzled-related protein (SFRP), the Wnt inhibitory factor 1 (WIF1), Cerberus, and the Dickkopf (DKK) family of secreted proteins [24]. Among them, the DKK family of Wnt antagonists has recently spurred an increased interest. The DKK family is comprised of four proteins (DKK1, DKK2, DKK3, and DKK4), which are synthesized as precursor proteins activated by proteolytic cleavage [25]. The DKK1 and DKK3 proteins are the most studied members of the family; these can inhibit Wnt signaling by binding to and degrading coreceptor LRP5/6 and thus have been considered as potential targets in diseases with an aberrant Wnt signaling activity [26, 27].

Of note, apart from its pivotal role in embryonic development and homeostatic self-renewal in adult tissues, Wnt signaling pathways exert both anti-inflammatory and proinflammatory functions, at least in part by interacting with the TLR/NF-*κ*B signaling pathway during inflammations [28, 29]. For example, the Wnt/*β*-catenin signaling is able to control inflammatory responses in infections caused by pathogenic bacteria [30]. In this regard, Wnt/*β*-catenin signaling is able to repress or enhance the NF-*κ*B signaling. And vice verse, the TLR pathway can either positively or negatively regulate Wnt/*β*-catenin signaling [28, 29, 31]. It has been recognized that the initiation and progression of COPD is in part attributed to chronic inflammation and continuing proteolysis of ECM and structural cell death, ultimately leading to an inability of the lung to activate self-repair mechanisms [32]. However, in IPF, the precise causes of pulmonary fibrosis are currently not fully elucidated, although the persistent or repetitive injury of lung is suggested as an etiology of pulmonary fibrosis. In this context, pulmonary inflammation has been recognized as a precursor to the development of fibrosis in several fibrotic lung diseases, such as Farmer's lung and bird fancier's lung [33]. Controversially, anti-inflammatory treatments are normally ineffective for IPF. The gene expression profile further reveals an elevated expression of genes associated with tissue remodeling, epithelial, and fibroblast genes in IPF lungs [34]. This controversial evidence leads the debate in the pathogenic roles of inflammation in IPF. Nonetheless, this paradoxical viewpoint of the role of inflammation in the pathogenesis of IPF has recently been revived, owing to recently licensed pirfenidone [35–38] and nintedanib [39], two novel agents with anti-inflammatory activity for IPF treatments that show promising therapeutic efficacy in clinical practice. In addition to a compelling body of evidence has historically suggested the involvement of immune system in pathogenesis of pulmonary fibrosis in both experimental models and humans [40, 41]. Together with the aforementioned regulatory roles of Wnt pathways in inflammation, Wnt signaling can exert as mediators or regulators of inflammatory responses in COPD and IPF.

## 3. Implications of Wnt Signaling in COPD and IFP

The lung is an organ continually undergoing epithelial injury caused by environmental insults from inhaled air, which can activate a variety of signaling pathways for injury repair to preserve its integrity [42]. There are increasing lines of evidence that suggest the involvement of Wnt signaling in lung development, homeostasis, and pathogenesis of pulmonary diseases, such as lung cancers and chronic pulmonary diseases, particularly the COPD, IPF, and asthma (Table 1). In this context, a dysregulated Wnt signaling activation may lead epithelial cells to aberrantly produce transforming growth factor (TGF-*β*) and to induce epithelial-to-mesenchymal transition (EMT) to regulate tissue repair and a deposition of extracellular matrix (ECM), such as fibronectin, matrix metalloproteinases (MMPs), and Snail [43]. Pathogenically, an aberrant Wnt signaling has been implicated in the development and progression of COPD and IPF, in which it leads to EMT, along with ECM deposition, pulmonary fibroblast proliferation and myofibroblast differentiation, and airway small muscle (ASM) cell proliferation and airway remodeling [10, 44]. Intriguingly, distinct Wnt/*β*-catenin signaling activation was observed in the lungs of both humans and animals in models of COPD and IPF. Specifically, a downregulated Wnt/*β*-catenin signaling was reported in smooth muscle cells of human small airway from COPD patients [14, 45], emphysematic lungs of murine model [14], and emphysematic patients [46, 47], while an enhanced canonical Wnt signaling was found in lungs of IPF patients and animals [10, 11, 48]. These clinical and experimental results may imply a distinct pathogenic role of Wnt/*β*-catenin signaling in COPD and IPF.

With respect to COPD, several lines of evidence have revealed an altered expression of Wnt signaling molecules (such as FZD4, Wnt 5A, and Wnt5B) in lung biopsies of COPD patients [46, 50, 56] and murine models [14]. Etiologically, cigarette smoking (CS) is one of the most important risk factors for COPD, which can directly alter lung tissue toward permanent airway remodeling [78]. Heijink et al. found that both transcriptional and translational expressions of Wnt4 were upregulated in primary bronchial epithelial cells (PBECs) from COPD patients relative to control nonsmokers [55]. Interestingly, CS led to decreased Wnt/*β*-catenin signaling activity in airway epithelia of COPD lung, as determined by transcripts of Wnt transcription factor TCF4 and Wnt receptor FZD4, which were negatively correlated with the smoking pack year and IL-1*β*, respectively [54]. Importantly, the suppression of FZD4 led to an impairment of Wnt/*β*-catenin-driven alveolar lung repair in AECII cells of COPD patients, smokers, or animal models [56]. In addition, a downregulated Wnt4 transcript but not the protein was also observed in PBECs of smoking COPD patients [55], and an enforced expression of Wnt4 could further increase cigarette smoke extract-(CSE-) induced proinflammatory cytokine release in bronchial epithelial cells [55]. These were inconsistent with a function of Wnt/*β*-catenin signaling in ECM production of pulmonary fibroblast and myofibroblast differentiation, suggesting an important role of the canonical Wnt pathway in regulating fibroblast phenotype and function in COPD [47, 79].

Interestingly, less abundant nuclear *β*-catenin-positive alveolar epithelial cells were observed in human COPD lungs as determined by an immunohistochemistry (IHC) assay in comparison with donor lungs, albeit no difference was detected in the mRNA expression profile of this key Wnt/*β*-catenin signaling components between COPD and donor lung homogenates [14]. Importantly, canonical Wnt signaling activity was decreased in experimental emphysema mouse models, and an activation of Wnt/*β*-catenin activation using lithium chloride (LiCl) led to an attenuation of experimental emphysema, as ascertained by an elevated expression of alveolar epithelial cell markers, decreased airspace enlargement, reduced collagen contents, and improved lung function [14]. Apart from Wnt/*β*-catenin signaling, noncanonical Wnt5A/B and their receptor FZD8 were upregulated or activated by TGF-*β*1 in pulmonary fibroblasts and lung tissues or CS from COPD patients, which impaired endogenous lung repair and induced inflammatory responses in COPD lung [46, 50, 53].

With respect to IPF, a compelling body of clinical evidence and experimental studies also demonstrated an aberrant Wnt signaling activation in IPF pathogenesis [9-11, 62, 63, 66, 67, 69], and RNA-Seq analysis of formalin-fixed, paraffin-embedded (FFPE) lung tissue from patients with IPF revealed enrichments of transcripts related to Wnt and TGF-*β* signaling pathways [80]. Blockades of the Wnt/*β*-catenin pathway showed an attenuated experimental fibrosis in mice [44, 81]. The perturbation of Wnt-pathway was directly correlated with abnormal myofibroblast activation [61], EMT [49, 82, 83] and fibrotic process amplification [84], although an increased expression of Wnt inhibitor DKK1 was found to predominantly localize in basal bronchial and hyperplastic alveolar epithelial cells of human IPF lung relative to transplanted donor lungs [60]. For example, an increased Wnt3A protein in fibrotic alveolar epithelia, accompanied by enhanced concentrations of interleukin 1*β* (IL-1*β*) and IL-6, was found in bronchoalveolar lavage fluid (BALF) from patients with IPF [67]. A clinical association study by Lam et al. further revealed that the expression of Wnt signaling coreceptors LRP5/6 of peripheral blood mononuclear cells (PBMCs) and Wnt signaling receptor FZD8 of IPF lung tissues were elevated in IPF patients, suggesting that they were independent factors associated with IPF disease progression and severity [66]. Experimentally, mice with deficient LRP5 or direct inhibition of *β*-catenin signaling by small molecular inhibitor showed a resistance to bleomycin-induced pulmonary fibrosis (PF), but failed to protect animals from PF induced by TGF-*β*, and transplantation of bone marrow cells of LRP5-deficient mice could not limit bleomycin-induced fibrosis in LRP5 wild-type mice. In addition, the lacking of LRP5 was associated with reduced TGF-*β* production in alveolar epithelial type 2 (AECII) cells and leukocytes [66]. Indeed, a recent study demonstrated that LRP5/*β*-catenin signaling controlled alveolar macrophage differentiation and inhibited bleomycin-induced murine pulmonary fibrosis [76]. LRP5-deficient mice exhibited reduced fibrosis with significantly fewer Siglec flow alveolar macrophages [76].

In addition, one of the characteristics of IPF is heterogeneous fibrosis with densely fibrotic areas surrounded by nonfibrotic, normal-looking tissues. Rydell-Tormanen et al. recently demonstrated a correlation between aberrant nonfibrotic parenchyma and the interaction of *β*-catenin inhibition, as well as Wnt5A/B activation in the lungs of IPF patients [9]. In this study, the expression of Wnt signaling molecules *β*-catenin, Wnt3A, inhibitor of *β*-catenin and T-cell factor (ICAT), Wnt5A/B, dishevelled associated activator of morphogenesis 1 (DAAM1), and nemo-like kinase (NLK) was examined in the lung parenchyma biopsies from 10 IPF patients and 7 healthy individuals. A significantly downregulated ICAT, but upregulated Wnt3A, *β*-catenin, Wnt5A/B, DAAM1, and NLK, was observed in normal-looking parenchyma of IPF lungs as compared with healthy lungs, suggesting an association of the degree of fibrosis and the interaction between *β*-catenin and Wnt5A/B in IPF [9]. An elevated Wnt5A [64, 82] and Wnt7B expression was also reported in human IPF lungs [62], and an expression of Wnt10A was correlated with a poor survival rate in the IPF [63]. Together with the aforementioned clinical and experimental evidences, these studies clearly suggest the important roles of Wnt signaling pathways in lung development injury repairs and pathogenesis of chronic pulmonary diseases. Therefore, numerous studies have started to interrogate molecular mechanisms underpinning the regulatory roles of Wnt signaling in the pathogenesis of COPD and IPF.

## 4. Molecular Mechanisms of Wnt Signaling in Pathogenesis of COPD

COPD is a devastating heterogeneous disease manifested with chronic airway inflammation, irreversible airflow limitation, accelerated distortion of normal lung architecture, and impairment of lung function [85, 86]. Phenotypically, it can be further characterized by two main intrapulmonary disorders: small airway disease and emphysema. Small airway disease is characterized by airway inflammation with increased mucus production and peribronchiolar fibrosis, and emphysema is defined as destruction of the alveolar surface area for gas exchange due to distal airspace enlargement [32]. Etiologically, primary causes of COPD include cigarette smoke (CS) and environmental factors in people with genetic predisposition [87]. Mechanistically, the initiation and progression of COPD is in part attributed to chronic inflammation and continuing proteolysis of ECM, structural cell death, and an inability of the lung to activate self-repair mechanisms [32].

Despite the increasing lines of evidence suggesting that Wnt/*β*-catenin signaling is implicated in pulmonary parenchymal tissue repair and airway epithelial cell differentiation, the underlying mechanisms of this pathway in small airway inflammation of COPD are far to be elucidated. By using human bronchial epithelial cells (16HBECs) and CS-induced COPD mouse models, Guo et al. recently found that *β*-catenin activator SB216763 and *β*-catenin small-interfering RNA (siRNA) could reduce and exacerbate the production of inflammatory cytokines TNF*α* and IL-1*β* in 16HBECs exposed to CS extract (CSE), respectively. Notably, *β*-catenin activator SB216763 significantly ameliorated peribronchial inflammatory cell infiltration, leukocyte influx, and the release of TNF*α* and IL-1*β* in the BALF of CS-exposed mice. Mechanistically, the SB216763-activated *β*-catenin could restore the CSE-repressed peroxisome proliferator-activated receptor (PPAR*δ*) and attenuate the CSE-induced phosphorylation of p38 mitogen-activated protein kinase (MAPK). By contrast, PPAR*δ* agonist was able to counteract *β*-catenin siRNA-mediated aggravation of phosphorylated p38 MAPK in response to CSE. Thus, an activation of PPAR*δ* or inhibition of p38 MAPK led a reduction of CSE-induced TNF*α* and IL-1*β* secretion in 16HBEC cells. This finding suggested that a repressed Wnt/*β*-catenin signaling might have an essential role in airway inflammation in COPD by promoting inflammatory cytokine production through a PPAR*δ*/p38 MAPK pathway in airway epithelial cells [54].

A dysregulated Wnt/*β*-catenin signaling is implicated in aberrant ECM deposition in chronic pulmonary diseases, such as COPD [47, 79]. Interestingly, growth factors and cytokines produced by epithelial cells and fibroblasts of COPD lung can influence Wnt/*β*-catenin signaling activity. For instance, basic fibroblast growth factor (bFGF) is a composition of mucus in COPD patients, which shows an ability to significantly induce the expression of *β*-catenin, Wnt5A, and RhoA in primary fibroblasts of rats COPD model, in comparison with fibroblasts from rats without COPD. As a consequence, the activated *β*-catenin is able to enhance the expression of *α*-smooth muscle actin (SMA) and fibronectin, accompanied with an increased ECM deposition and differentiation of pulmonary fibroblasts and myofibroblasts in COPD rat. Of note, *α*-SMA plays a critical role in airway remodeling of COPD lung. Previously, studies demonstrated that aforementioned bFGF-induced *α*-SMA and fibronectin could be abrogated by siRNA to *β*-catenin, Wnt5A, or RhoA transduced by adenoviral vectors in primary fibroblasts of rats, indicating a pivotal role of the *β*-catenin-dependent canonical Wnt pathway and Wnt5A-mediated noncanonical Wnt signaling in mediating fibroblast morphology and function in pathogenesis of COPD [88]. In addition to Wnt/*β*-catenin, Wnt5A-activated noncanonical Wnt pathways also play a critical role in TGF-*β*-induced ECM expression in airway smooth muscle cells and promote tissue fibrosis, where Wnt5A can be targeted by TGF-*β* and engaged *β*-catenin-independent noncanonical Wnt signaling by activating Ca2-NFAT and JNK to induce expression of ECM genes [89].

Apart from a repressed *β*-catenin-dependent Wnt canonical pathway that was linked to impaired lung repair in COPD, an enhanced *β*-catenin-independent noncanonical Wnt signaling was also observed in the lungs from COPD patients and in pulmonary fibroblasts exposed to COPD-related stimuli, such as TGF-*β*, CS, and cellular senescence. In this regard, Wnt5A and Wnt5B are ligands that mediated noncanonical Wnt signaling pathways [90]. Importantly, a shift of Wnt signaling from canonical to noncanonical pathways has been suggested to contribute to COPD pathogenesis, particularly in emphysema [46]. In this context, an overexpression of mature lung-specific Wnt5A was found to functionally attenuate canonical Wnt-driven alveolar epithelial cell wound healing and transdifferentiation in vitro, which in turn exacerbate airspace enlargement in elastase- or CS-induced experimental emphysema in vivo [46]. In contrast, an inhibition of Wnt5A led an attenuation of lung tissue destruction, improvement of lung function, restoration of Wnt canonical signaling target gene expression, and alveolar reepithelialization in murine COPD models [46].

Similar to what is seen in Wnt5A and emphysema, an exposure of CS to PMBC cells of COPD patients was found to induce Wnt5B expression, along with an increased expression of genes related to small airway remodeling, such as fibronectin, matrix MMP-2, MMP-9, and Snail [50]. Mechanistic analysis further revealed a TGF-*β*/Smad3 signaling-mediated augmentation of small airway remodeling related genes in BEAS-2B cells that exaggerated expression of Wnt5B. Such an upregulated Wnt5B and remodeling-related gene expression were also observed in the air-liquid interface- (ALI-) cultured PBEC cell model, particularly in the cells from COPD patients [50]. In line with this finding, van Dijk et al. recently demonstrated that Wnt5B induced inflammatory responses in human lung fibroblasts [53]. In this study, the authors ascertained inflammatory cytokine releases of human lung fibroblasts, and their results showed that Wnt5B had an ability to induce CXCL8 release in MRC-5 human fibroblasts upon Wnt5A/B simulation. Equally noteworthy, Wnt5B induced more IL-6 and CXCL8 release in fibroblasts from COPD patients relative to non-COPD controls [53]. Mechanistically, these were primarily mediated by the FZD2 and TAK1 signaling and the activation of JNK, p38, and p65 NF-*κ*B signaling, but without an involvement of *β*-catenin signaling. Of interest, the Wnt5B-induced production of IL-6 and CXCL8 could be reduced when JNK and p38 signaling was inhibited [53]. Together with the aforementioned findings [50, 53], these studies suggest that Wnt5B-mediated noncanonical Wnt signaling may exert a detrimental effect in COPD patients to promote small airway remodeling, which may represent a therapeutic target for COPD treatment [53].

## 5. Molecular Mechanisms of Wnt Signaling in the Pathogenesis of IPF

Idiopathic pulmonary fibrosis (IPF) is a chronic interstitial lung disease with unknown etiology. This debilitating disease is characterized by the loss of normal respiratory surface area, with alterations of alveolar epithelium, myofibroblast activation, and increased extracellular matrix deposition [91, 92]. Due to the lacking efficacy of anti-inflammatory therapy, the mostly acceptable hypothesis for pathogenesis of IPF is continuous alveolar epithelium microinjuries and abnormal tissue repairs caused by a cascade of dysregulated signaling in epithelial-fibroblast crosstalk [93], albeit inflammations have been historically suggested to play a central role in the pathogenesis of this untreatable disease. In this regard, the cooperation between TGF-*β* and Wnt signaling has been recognized to play a key role in the development, differentiation, and EMT in mouse model of IPF [10, 94].

Indeed, Wnt/*β*-catenin signaling is one of mediators linked to the impaired wound-healing response in pulmonary fibrosis (PF), in which the crosstalk between this signaling and TGF-*β* is crucial for EMT and the development of IPF [9, 63, 71]. In this regard, activation of *β*-catenin has been recognized as a key event in IPF [10]. Activated Wnt/*β*-catenin signaling could induce AECII cells to produce IL-1*β* in PF, in which AECII cells are a relevant source of proinflammatory cytokines induced by *β*-catenin activation [67]. Interestingly, both IL-1*β* and IL-6 were significantly induced in mice lungs intratracheally instilled with bleomycin or Wnt3A protein in vivo and in primary murine AECII cells exposed to Wnt3A protein in vitro [67]. Wnt10A is another ligand involved in the Wnt/*β*-catenin signaling pathway and has been demonstrated to correlate with a poor survival in IPF patients [63]. Experimental pathogenic studies further revealed that an induced Wnt10A expression correlated with an enhanced TGF-*β* in the lung of bleomycin-induced PF mice. Intriguingly, the introduction of TGF-*β* could induce expression of Wnt10A and collagen. In contrast, siRNA-mediated inhibition of Wnt10A reduced the production of collagen in fibroblasts cells, suggesting an important pathogenic role of Wnt10A/TGF-*β* signaling activation in IPF [63].

Furthermore, a recent study demonstrated that TGF-*β* could induce expression of extracellular matrix metalloproteinase inducer (EMMPRIN) in AECII cells, which in turn activated the Wnt/*β*-catenin signaling pathway. The activated Wnt/*β*-catenin signaling was found to contribute the persistent fibroproliferative state seen in IPF, by inducing antiapoptotic and profibrotic phenotypes in lung fibroblasts, sequentially promoting proliferation, and survival and differentiation of myofibroblasts to pulmonary fibroblasts [95]. Such a crosstalk between Wnt/*β*-catenin signaling and TGF-*β* is critical for regulating EMT, an important mechanism involved in epithelial repair and IPF pathogenesis, in which TGF-*β* and Smad2/3 can activate Wnt/*β*-catenin signaling [94]. Mechanistically, the presence of JNK1 was in part required for Wnt/*β*-catenin signaling-induced EMT of lung epithelial cells [83]. In addition, the TGF-*β*-activated Wnt/*β*-catenin signaling could be directed via a PI3K/PKB/GSK3-dependent effect on the stability of *β*-catenin protein and ERK1/2-dependent effects on a canonical Wnt signaling-dependent *β*-catenin gene expression [58]. Indeed, previous studies have revealed that the Wnt pathway inhibitor DKK1 showed no effect on TGF-*β*-induced ECM protein production or *β*-catenin expression in human airway smooth muscle cells [89]; TGF-*β* failed to promote the expression of canonical Wnt target genes in human lung fibroblasts [47]. These findings further supported that the TGF-*β*/Smad signaling-induced Wnt *β*-catenin activation might not be dependent on canonical Wnt signaling [58]. However, activating Wnt/*β*-catenin signaling was required for TGF-*β*/Smad2/3 signaling during myofibroblast proliferation, suggesting that Wnt/*β*-catenin-activated TGF-*β*/Smad2/3 signaling in epithelial and mesenchymal cells contributed to lung fibrosis [68]. Consistently, previously experimental studies in murine lung epithelial cells also demonstrated that activating the Wnt/*β*-catenin pathway could lead to the expression of EMT-related genes, CArG box-binding factor-A, fibroblast-specific protein (FSP)-1, *α*-SMA, and vimentin, but a concomitant loss of zona occludens-1 (ZO-1). In contrast, siRNA-mediated knockdown of *β*-catenin or JNK1 led a largely abolished Wnt3A, *β*-catenin, and dominant active S37a-*β*-catenin-induced EMT in murine lung epithelial cells [83].

Interestingly, TGF-*β* also demonstrated an ability to stabilize *β*-catenin [96], leading to increased Wnt5A expression and subsequent ECM production in ASM cells [89, 97]. In this context, TGF-*β*/Smad signaling could activate Wnt/*β*-catenin through a mechanism by which Smad3 directly interacted with *β*-catenin. Of note, ICAT, a Wnt/*β*-catenin inhibitor that functions by dissociating the interaction between *β*-catenin and different transcription complexes [94] could abrogate the interaction between TGF-*β* and *β*-catenin [98]. In addition to TGF-*β*, Wnt5A also has the capacity to stabilize *β*-catenin [4, 9].

Therefore, consistent with the Wnt/*β*-catenin canonical pathway, the crosstalk between TGF-*β* and *β*-catenin-independent Wnt signaling clearly has important implications in the pathogenesis of IPF. This was further supported by evidence of elevated Wnt5A in pulmonary fibroblasts of IPF patients. In this scenario, it might function to promote proliferation and survival of lung fibroblasts as well as augment expression of ECM, such as fibronectin and integrin of lung cells [82]. In this context, Wnt5A was expressed in pulmonary epithelial cells, smooth muscle cells, endothelial cells, and myofibroblasts of fibroblastic foci and throughout the interstitium of IPF lung. Similarly, an abundance of the Wnt7B protein was found in regions of active hyperplasia, metaplasia, and fibrotic change in human IPF lungs. This suggests that Wnt7B is related to the pathogenesis of IPF [62]. Indeed, an enforced expression of Wnt7B significantly led to an induction of TGF-*β*-independent Wnt5A protein in normal human smooth muscle cells and fibroblasts, albeit TGF-*β* itself could also induce Wnt5A expression in IPF lung fibroblasts. However, the overexpressed Wnt7B failed to increase the abundance of Wnt5A protein in IPF myofibroblasts, partially owing to Wnt5A being already highly expressed in these cells [64]. These studies imply that an activated TGF-*β* and Wnt7B signaling controls Wnt5A expression in pulmonary fibroblasts of IPF lung, which may represent key signaling pathways that modulate the pathogenesis of IPF [64].

In addition to Wnt5A, Wnt5B was also highly expressed in human lung fibroblasts, which was TGF-*β*/Smad3 dependent. It could also mimic functional effects of TGF-*β* on induction of fibroblast activation by enhancing the expression of ECMs, including fibronectin, versican, *α*-SMA, and connective tissue growth factor (CTGF) [47]. Interestingly, a recent study demonstrated that TGF-*β*-induced profibrotic signaling was partially regulated by the Wnt receptor FZD8 through a Smad3-dependent signaling; knockdown of FZD8 expression abolished TGF-beta1-induced production of ECM and partially prevented Wnt5B-induced alterations in cellular impedance in lung fibroblasts [58]. Even more, FZD8 gene null mice exhibited an attenuated bleomycin-induced lung fibrosis phenotype in vivo [58]. Given that TGF-*β* is a key mediator in the development and progression of IPF and the importance of interactions between TGF-*β*/Smad and Wnt signaling in IPF pathogenesis, this study indicated that Wnt5B engaged FZD8 receptor to activate noncanonical Wnt signaling, which sequentially exerted a regulatory role in TGF-*β*/Smad-induced effects on fibroblast activation [58].

Taken together, increasing clinical and experimental evidences have demonstrated several important roles of Wnt signaling in the pathogenesis of chronic pulmonary diseases, particularly in COPD and IPF. As a result, it is a rationale to interrogate therapeutic potentials by targeting Wnt signaling pathways for COPD and IPF treatments.

## 6. Wnt Signaling Pathways as Targets for COPD and IPF Treatments

Great implications in the development and progression of COPD and IPF have led to explore the potential of targeting Wnt signaling pathways for treatments of these diseases. Indeed, several lines of evidence have demonstrated that Wnt/interleukin and/or Wnt/TGF-*β* signaling axes may represent novel useful therapeutic targets for treatment options in patients with COPD or IPF.

With respect to IPF, aberrant activation of Wnt and inflammation pathway leads to the perturbation of profibrotic effects [67]; inhibiting the Wnt/*β*-catenin signaling cascade could reduce bleomycin-induced murine pulmonary fibrosis model [61, 71, 99, 100]. *β*-catenin is the key mediator of the canonical Wnt signaling pathway; therefore, *β*-catenin was targeted using several strategies in bleomycin-induced PF murine models. For instance, the CREB-binding protein (CBP) and its homologue p300 are transcriptional coactivators of *β*-catenin. An interaction between CBP/*β*-catenin is associated with proliferation, whereas an interaction between p300/*β*-catenin was the first essential step to initiate differentiation [65]. Therefore, an aberrant use of these two transcriptional programs by *β*-catenin may be responsible for the improper wound healing process, which may be important in the pathogenesis of pulmonary fibrosis [65, 101]. Mice that concurrently received bleomycin and ICG-001, a small molecule inhibitor that selectively blocks CBP/*β*-catenin without interfering p300/*β*-catenin interaction, showed a significantly inhibited *β*-catenin signaling and attenuated bleomycin-induced pulmonary fibrosis with preserved epithelium in the lung. Furthermore, later administrations of ICG-001 in bleomycin-injured mice also halted disease progression, reversed established injury, and significantly improved survival rates [65].

Similarly, inhibiting Wnt/*β*-catenin signaling using small interfering RNA (siRNA) [61] or short hairpin RNA (shRNA) [102] to *β*-catenin gene also indicated abilities to attenuate pulmonary fibrosis in mice. In one study, C57BL/6N mice were first intratracheally instilled with bleomycin to generate pulmonary fibrosis, then administered with siRNA in the trachea to inhibit Wnt/*β*-catenin signaling two hours before the bleomycin instillation and then every 2 days for 14 days until the end of experiment. The administration of siRNA resulted in a significant inhibition of Wnt/*β*-catenin, a remarkable decrease in MMP2 and TGF-*β* expression in lung tissues, as well as an attenuated bleomycin-induced pulmonary fibrosis in murine models [61]. In line with this finding, Wang et al. examined the potential of lentivirus-mediated shRNA to *β*-catenin in halting silica-induced lung fibrosis in mice [102]. The intratracheal administration of lentiviral *β*-catenin shRNA could significantly attenuate silica-induced pulmonary fibrosis as evidenced by hydroxyproline content and collagen I\III synthesis, along with a notable reduction of *β*-catenin expression in the lung and TGF-*β* abundance in BALF of silica-administrated mice [102].

In addition, XAV939 is an extensively investigated Wnt/*β*-catenin signaling inhibitor that targets tankyrase 1/2, which also showed the ability to attenuate bleomycin-induced lung fibrosis, improve the survival of mice with lung injuries, and reduce Wnt/ *β*-catenin signaling activity [100, 103]. Interestingly, XAV939 also could also promote the differentiation of bone marrow-derived mesenchymal stem cells (BM-MSCs) into epithelium-like phenotype, but inhibit the proliferation and myofibroblast differentiation of NIH/3T3 fibroblasts in a coculture model of BM-MSCs and NIH/3T3 fibroblasts [100].

Another potential IPF treatment, aside from blocking Wnt/*β*-catenin pathways by directly targeting *β*-catenin, could be to target other signaling molecules in this signaling cascade. For example, the expression of WNT1-inducible signaling protein-1 (WISP1), a Wnt target gene, was elevated in AECII cells of both a mouse model of pulmonary fibrosis and in IPF patients [48]. Functionally, WISP1 could mediate IL-6-dependent cell proliferation through a mechanism orchestrated by a variety of profibrotic mediators, including Wnts, TGF-*β*1, and TNF-*α* in primary human lung fibroblasts [104]. Cell exposed to recombinant WISP1 led to an increased proliferation and EMT in mouse primary AECII cells, as well as an increased deposition of ECM components in mouse and human lung fibroblasts. More importantly, neutralizing WISP1 with specific antibodies led to the attenuation of lung fibrosis and increased survival rate in bleomycin-induced PF mice, along with a reduced collagen deposition and expression of genes associated with EMT [48]. Targeted blocking of disheveled (Dvl), a key component of Wnt/*β*-catenin signaling, is another example. NSC668036, a small organic inhibitor of the PDZ domain of Dvl was able to suppress the expression of Wnt/*β*-catenin signaling and abrogate TGF-*β*1-induced cell migration and ECM production in fibroblasts in vitro. Furthermore, it also displayed the ability to suppress TGF-*β*1 expression and ECM deposition, while enhancing the expression of cytokeratin (CK)19, occludin, and E-cadherin genes to inhibit pulmonary fibrogenesis in bleomycin-induced PF mice [59]. Of note, Wnt/*β*-catenin pathway blocking-mediated attenuation of bleomycin-induced PF was accompanied by a decreased expression of TGF-*β*1 and fibroblast growth factor (FGF) 2 in vitro and in vivo. These results support that controlling the aberrant expression of TGF-*β*1 and FGF2 via inhibition of Wnt/*β*-catenin signaling could serve as a potential therapeutic strategy for PF [71]. Collagen triple helix repeat containing 1 (CTHRC1) protein showed the potential to inhibit Smad2/3 activation and alleviate bleomycin-induced fibrosis in mice [105]. Immunohistochemistry analysis demonstrated that mice-deficient Cthrc1 lung sections showed intracellular localization and activation of *β*-catenin Y654 in areas of tissue remodeling, suggesting CTHRC1 might target *β*-catenin signaling [105]. These studies thus support the concept of inhibition of the Wnt/*β*-catenin signaling pathway for the treatment of IPF.

Interestingly, however, with regards to COPD, a reactivation of Wnt/*β*-catenin signaling pathways is a potential option for treatments, since its activity was repressed in human lung samples of patients with COPD [14, 50, 55]. Particularly, the potential of LiCl, a GSK3*β* inhibitor that activates Wnt/*β*-catenin pathways in the prevention or attenuation of airspace enlargement in CS-induced emphysema model, supports the protective role of Wnt signaling from the development of emphysema [14]. This finding was recently further confirmed by Uhl et al. in murine-derived three-dimensional (3D) ex vivo lung tissue culture model, which was generated from emphysematous mice 7 days postelastase treatment and characterized by reduced inflammatory processes and established airspace enlargement [51]. Pharmacological inhibition of GSK3*β* using LiCl or CHIR 99021 (CT) led to the reactivation of Wnt/*β*-catenin signaling, which in turn altered macrophage activity and elastin remodeling. Furthermore, there was an increased expression of active *β*-catenin protein, Wnt/*β*-catenin target genes, and alveolar epithelial cell markers, but a reduction in MMP12 in this 3D-LTC model [51]. Of further note, most recent studies by Baarsma et al. suggested that inhibiting Wnt5A led a reactivation of Wnt/ *β*-catenin in the lungs of COPD murine models, suggesting that it was a potential target for COPD treatment target [46, 57].

## 7. Conclusions

COPD and IPF are chronic and progressive diseases that severely affect lung functions. They are caused by a combination of environmental factors and genetic predeposition in elderly populations, indicated by a progressive loss of alveolar parenchyma and severe respiratory impairment. However, they are recognized as two distinct chronic pulmonary disorders with unique clinical and pathological features [12]. Pathogenically, COPD and IPF are the result of aberrant lung injury repair caused by dysregulation of signaling pathways involved in lung tissue regeneration. In particular, this involves developmental signaling such as Wnt and Notch. These eventually result in aberrant pulmonary inflammation, tissue remodeling, and functional impairment in alveolar parenchyma (i.e., irreversible fibrosis and bronchiolar honeycombing in IPF, emphysema, airway inflammation, and remodeling in COPD) [12]. Among these developmental pathways, aberrant Wnt signaling is an important implication in the pathogenesis of these incurable diseases. It plays distinct pathogenic roles in COPD and IPF through mechanisms interacting with other transcriptional factors and growth factor signaling, such as TGF-*β* and FGF.

**Figure 2:**
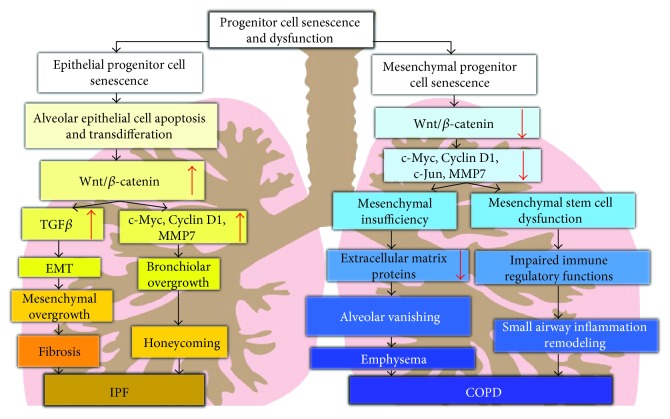
Scheme of possibly distinct pathogenic role of Wnt/*β*-catenin signaling in COPD and IPF. Possible mechanisms of Wnt/*β*-catenin signaling in the pathogenesis of COPD (right) and IPF (left) are proposed based on the literatures. In IPF, an enhanced Wnt/*β*-catenin signaling of epithelial cells interacts with TGF-*β* to promote EMT, which leads to mesenchymal overgrowth and fibrosis. On the other hand, activated Wnt signaling can increase the expression of Wnt target genes related to cell proliferation and ECM, which leads to bronchiolar overgrowth and honeycombing. In the case of COPD, a decreased Wnt/*β*-catenin signaling activity in the mesenchymal compartment results in the reduced expression of Wnt target genes related to cell proliferation, leading to mesenchymal deficiency and the reduction of ECM protein expression in alveolar cells, accordingly the development of emphysema or dysfunctions MSCs and immune regulations in small airway, which leads to enhance inflammation in airway.

Both environmental and genetic factors underlie abnormal renewal of epithelial or mesenchymal progenitor cells, which may induce either epithelial progenitor cell senescence or mesenchymal progenitor senescence. In this respect, aberrant Wnt signaling may be involved in the onset and progression of diseases in cell-type and activity-dependent manners (i.e., an enhanced Wnt/*β*-catenin signaling in epithelial cells favors for pathogenesis of IPF and a diminished Wnt/*β*-catenin signaling in mesenchymal compartment may promote COPD progression) (Figure 2). This view is supported by clinical and experimental evidences of activated Wnt/*β*-catenin signaling in IPF lung epithelial cells and fibroblasts, in particular during the process of epithelial cell injury and repair in vitro and in vivo. This notion can be further supported by amelioration of fibrosis and improved survival in bleomycin-induced PF mice that were administrated ICG-001 and XAV939, pharmacological inhibitors of Wnt signaling. Further, a decreased Wnt/*β*-catenin is detected in COPD lung specimens, particularly in experimental emphysema. Most importantly, *β*-catenin activator SB216763 shows an ability to reduce peribronchial inflammatory cell infiltration and concentrations of TNF*α* and IL-1*β* in BALF of CS-exposed mice. Similarly, activating Wnt/*β*-catenin signaling using recombinant Wnt ligand proteins, Wnt signaling activator LiCl, and HC also showed the ability to enhance epithelial cell proliferation and survival, subsequently lead the prevention or attenuation airspace enlargement with restored alveolar epithelial structure and function in emphysema models.

Undoubtedly, these promising experimental results strongly support that the Wnt signaling pathway plays an important role in pathogenesis of COPD and IPF. As a result, this pathway should be targeted in pharmacological interventions to attenuate these devastating diseases. However, although it is a key pathway involved in cell proliferation, stem cell fate determination and tumorigenesis, molecular mechanisms underpinning the pathogenesis of Wnt signaling in both COPD and IPF, remain largely unknown. Therefore, further studies are required to understand mechanistic principles underlying the role and function of Wnt pathways in pulmonary injury repair and pathogenesis of COPD and IPF.

## Figures and Tables

**Table 1:** Wnt signaling involved in the pathogenesis of COPD and IPF.

Disease	Wnt signaling	Model	Function/mechanism	Ref.
COPD	Wnt/*β*-catenin and PKC signaling	HBEC cells	An interaction of Wnt/*β*-catenin and PKC signaling reduced nicotine-induced surfactant protein A (SPA) SPD: surfactant protein D (SPD) in HBEC cells	[49]
COPD	Wnt5B	BEAS-2B and PBEC cells	An exaggerated Wnt5B expression upon cigarette smoke exposure led to TGF-*β*/Smad3-dependent expression of genes related to airway remodeling in the bronchial epithelium of COPD patients	[50]
COPD	Wnt/*β*-catenin	3D cultures of murine and patient-derived lung tissue cultures (LTCs)	Enhanced Wnt/*β*-catenin signaling using GSK3*β* inhibitor LiCl and CHIR 99021 attenuated pathological features of COPD patient-derived 3D-LTCs	[51]
COPD	Wnt/*β*-catenin signaling	Human primary pulmonary fibroblasts derived from non-COPD individuals	Wnt/*β*-catenin signaling contributes to ECM production and differentiation pulmonary fibroblasts by pulmonary fibroblasts	[47]
COPD	Wnt3a/*β*-catenin signaling pathway	HBEC cells	Wnt3a/*β*-catenin signaling promotes HBEC cells undergoing EMT upon nicotine stimulation in vitro	[52]
COPD	Noncanonical Wnt5B signaling	Human primary pulmonary fibroblasts derived from COPD patients and non-COPD individuals	Wnt5B induces IL-6 and CXCL8 secretion in pulmonary fibroblasts through FZD2 receptor and TAK1 signaling	[53]
COPD	Wnt/*β*-catenin signaling	HBEC cells, mouse lung tissues, and mouse models	Wnt/*β*-catenin has an essential role in airway inflammation of COPD by PPAR*δ*/p38 MAPK pathway, a cigarette smoke reduced this signaling activation which promotes inflammatory cytokine production in airway epithelium	[54]
COPD	Wnt/*β*-catenin signaling	Murine emphysema models	A decreased Wnt/*β*-catenin signaling activity is involved in parenchymal tissue destruction and impaired repair capacity in lung of murine emphysema model	[14]
COPD	Wnt/*β*-catenin signaling	Human lung epithelial cell lines and PBEC cells	Wnt4 expression is downregulated in airway epithelial cells exposed to cigarette smoke extract (CSE), which in turn induces proinflammatory cytokine release of cells	[55]
COPD	FZD4, Wnt/*β*-catenin signaling	Lung tissues and primary AECII cells of COPD patients and smokers, mouse emphysema models	Reduced expression of FZD4 prevents Wnt/*β*-catenin-driven alveolar lung repair in COPD	[56]
COPD	Wnt5A	Elastase and CS-induced COPD murine models	An inhibition of Wnt5A-mediated noncanonical Wnt signaling leads the attenuation of lung tissue destruction, improvement of lung function, and restoration of expression of Wnt/*β*-catenin signaling target genes in the elastase and CS-induced COPD models	[46, 57]
COPD	Noncanonical Wnt signaling	FZD8-deficient mice, primary human lung fibroblasts, and primary human airway epithelial cells	FZD8 receptor is associated with chronic bronchitis and is involved in cytokine secretion from human pulmonary fibroblasts as well as acute CS-induced inflammation in mice	[58]
IPF	Wnt/*β*-catenin signaling and TGF-*β*	Pulmonary fibroblasts, bleomycin-induced pulmonary fibrosis murine model	An inhibition of Wnt/*β*-catenin signaling by targeting Dvl leads an effective alleviation of fibrotic lung diseases in mice.	[59]
IPF	Wnt/*β*-catenin, DKK1, DKK4	Human bronchial and alveolar epithelial cell lines/bronchoalveolar tissues	DKK1 and DKK4 proteins are expressed in human IPF lung epithelia; Wnt/*β*-catenin-induced epithelial cell proliferation can be regulated by DKK1 in a dose-dependent fashion	[60]
IPF	Wnt/*β*-catenin signaling pathway	C57BL/6N mice	Blockade of the Wnt/*β*-catenin signaling cascade attenuates bleomycin-induced PF in mice	[61]
IPF	Wnt7B	Human lung tissue samples	Wnt7B is expressed at high concentrations in regions of active hyperplasia, metaplasia, and fibrotic change of lungs in IPF patients	[62]
IPF	Wnt10A	Beomycin-induced mouse PF model	Wnt10A activates TGF-*β* signaling, which plays an important role in the pathogenesis of IPF via TGF-*β* activation, suggesting it may be a sensitive predictor for the onset of human IPF	[63]
IPF	Wnt/*β*-catenin signaling, Wnt5A/B	Human diagnostic biopsies/donated lungs	An interaction between the inhibition of *β*-catenin signaling and activation of Wnt5A/B is correlated with aberrant nonfibrotic parenchyma in IPF lungs	[9]
IPF	Wnt5A	IPF or UIP tissue sections	A wide distribution of Wnt5A is expressed in cells of IPF lung, which can be significantly induced by Wnt7B and TGF-*β*1	[64]
IPF	Wnt1inducible signaling protein-1 (WISP1)	A mouse model of pulmonary fibrosis, AECII cells	WISP1 is a key regulator of AECII cell hyperplasia in pulmonary fibrosis of murine models	[48]
IPF	Wnt/*β*-catenin/CREB binding protein (CBP) signaling	Bleomycin-induced lung fibrosis in mice	ICG-001 is selective blockade for *β*-catenin/CBP, which shows ability to prevent fibrosis when it is concurrent with bleomycin and reverse established fibrosis and significantly improve survival of bleomycin-induced lung fibrosis mice	[65]
IPF	Wnt/*β*-catenin signaling	Bleomycin-induced lung fibrosis mice	Wnt coreceptor, LRP5, is a genetic driver of lung fibrosis in bleomycin-induced lung fibrosis mice, which can be served as a marker of disease progression and severity in patients with IPF	[66]
IPF	Wnt coreceptor LRP5	Primary murine AECTII cells	Revealed that the alveolar epithelium is a relevant source of proinflammatory cytokines induced by active Wnt/*β*-catenin in pulmonary fibrosis	[67]
IPF	Wnt/*β*-catenin signaling	A549 cell line and bleomycin-induced pulmonary fibrosis Sprague Dawley rat model	Wnt/beta-catenin signaling activates TGF-beta/Smad2/3 signaling for myofibroblast proliferation in vitro and in vivo	[68]
IPF	Wnt/*β*-catenin signaling	Human lung biopsies from IPF patients and non-IPF individuals	Single cell RNA-Seq revealed aberrant canonical Wnt signaling activation in IPF	[69]
PF	Wnt/*β*-catenin signaling	Coculture of BM-MSCs and AECII cells, NIH/3T3 fibroblasts	Wnt/*β*-catenin signaling inhibitor XAV939 is able to promote the differentiation of BM-MSCs into an epithelium-like phenotype in the coculture system, which also shows a capacity to inhibit the proliferation and myofibroblast differentiation of NIH/3T3 fibroblasts	[61]
PF	Wnt/*β*-catenin signaling	MLE-12 cells	Regulatory T-cell- (Treg-) promoted EMT of MLE-12 cells is mediated by Wnt/*β*-catenin signaling	[70]
PF	Wnt/*β*-catenin signaling	Bleomycin-induced murine fibrotic lung tissue and primary AECII cells	An inhibition of Wnt/*β*-catenin signaling suppressed the aberrant expression of TGF-*β*1 and FGF2 in bleomycin-induced murine fibrotic lung tissues and primary AECII cells, suggesting Wnt/*β*-catenin pathway can be served as a potential therapeutic strategy for PF	[71]
PF	Wnt antagonists, SFRP1, and FRZB	Alveolar epithelial cell line, lung fibroblast cell line, and bleomycin-induced lung fibrosis murine model	SFRP1 counteracts the effect of TGF-*β*1in pulmonary cells in vitro, but loss of neither SFRP1 nor FRZB alters fibrotic outcomes in the lungs in mice	[72]
PF	Wnt/*β*-catenin signaling	BALB/c mice	An upregulation of *β*-catenin and elevation of TGF-*β*1 are associated with PTX-mediated transformation of pulmonary emphysema into pulmonary fibrosis under chronic a CS exposure	[73]
PF	Wnt/*β*-catenin signaling	HCl-induced acute lung injury SD rats	The aberrant activation of Wnt/*β*-catenin signaling induces the myofibroblast differentiation of engrafted MSCs in HCl-induced acute lung injury SD rats	[74]
PF	Wnt/*β*-catenin signaling	Primary lung microvascular endothelial cells, bleomycin-induced lung fibrosis mice	Repeated systemic administrations disrupt a normally fine-tuned balance in the Wnt signaling and induce reactive oxygen species (ROS) to cause DNA damage and fibrosis partially be affecting the endothelial niche	[75]
PF	LRP5, Wnt/*β*-catenin signaling	Bleomycin-induced lung fibrosis mice, LRP5-deficient mice	LRP5/*β*-catenin signaling controls alveolar macrophage differentiation and inhibits resolution of pulmonary fibrosis	[76]
PF	Wnt/*β*-catenin signaling	Human IMR-90 lung fibroblast cells	TGF-*β*1 can activate Wnt/*β*-catenin signaling pathway	[77]

3D-LTCs: three-dimensional lung tissue cultures; AEC II: alveolar epithelial cell type II; ASM: airway smooth muscle; APC: adenomatous polyposis coli; BALF: bronchoalveolar lavage fluid; BM-MSCs: bone marrow-derived mesenchymal stem cells; CBP: CREB-binding protein; COPD: chronic obstructive pulmonary disease; CS: cigarette smoke; CSE: cigarette smoke extract; CTGF: connective tissue growth factor; CXCL: chemokine (C-X-C motif) ligand; DAAM1: dishevelled associated activator of morphogenesis 1; DKK: Dickkopf; Dvl: disheveled; ECM: extracellular matrix; EMMPRIN: extracellular matrix metalloproteinase inducer; EMT: epithelial-mesenchymal transition; FEV1: forced expiratory volume in 1 second; FGF: fibroblast growth factor; FRZB: frizzled-related protein; FZD: frizzled; GSK: glycogen synthase kinase; HBECs: human bronchial epithelial cells; hBSMC: human bronchial smooth muscle cell; IL: interleukin; IPF: idiopathic pulmonary fibrosis; JNK: c-Jun N-terminal protein kinases; LRP: low-density lipoprotein-related receptor; MLE: mouse lung epithelial; MMP: matrix metalloproteinase; NFAT: nuclear factor of activated T-cells; NF-*κ*B: nuclear factor-*κ*B; NLK: nemo-like kinase; PBEC: primary bronchial epithelial cell; PBMC: peripheral blood mononuclear cell; PF: pulmonary fibrosis; PKC: protein kinase C; PSMCs: parabronchial smooth muscle cells; PTX: pentoxifylline; Ror: receptor tyrosine kinase-like orphan receptor; RYK: receptor-like tyrosine kinase; SD: Sprague Dawley; SFRP1: secreted frizzled-related protein-1; shRNA: short hairpin RNA; siRNA: small interfering RNA; SMA: smooth muscle actin; SPA: surfactant protein A; SPD: surfactant protein D; TAK1: transforming growth factor-*β* activated kinase-1; TCF/LEF1: T-cell factor/lymphoid enhancer factor 1; TGF-*β*: transforming growth factor-beta; TNF*α*: tumor necrosis factor *α*; Th2: helper 2 T-cells; UIP: usual interstitial pneumonia; WIF1: Wnt inhibitory factor 1; WISP1: Wnt1 inducible signaling pathway protein-1; Wnt: wingless-type MMTV integration site.

## Abbreviations

3D-LTCs: Three-dimensional lung tissue cultures
AEC II: Alveolar epithelial cell type II
APC: Adenomatous polyposis coli
ASM: Airway smooth muscle
BALF: Bronchoalveolar lavage fluid
BM-MSCs: Bone marrow-derived mesenchymal stem cells
CBP: CREB-binding protein
COPD: Chronic obstructive pulmonary disease
CS: Cigarette smoke
CSE: Cigarette smoke extract
CTGF: Connective tissue growth factor
CTHRC1: Collagen triple helix repeat containing 1
CXCL: Chemokine (C-X-C motif) ligand
DAAM1: Dishevelled associated activator of morphogenesis 1
DKK: Dickkopf
Dvl: Disheveled
ECM: Extracellular matrix
EMMPRIN: Extracellular matrix metalloproteinase inducer
EMT: Epithelial-mesenchymal transition
FEV1: Forced expiratory volume in 1 second
FGF: Fibroblast growth factor
FRZB: Frizzled-related protein
FZD: Frizzled
GSK: Glycogen synthase kinase
HBECs: Human bronchial epithelial cells
hBSMC: Human bronchial smooth muscle cell
IL: Interleukin
IPF: Idiopathic pulmonary fibrosis
JNK: c-Jun N-terminal protein kinases
LRP: Low-density lipoprotein-related receptor
LTRC: Lung Tissue Research Consortium
MLE: Mouse lung epithelial
MMP: Matrix metalloproteinase
NFAT: Nuclear factor of activated T-cells
NF-*κ*B: Nuclear factor-*κ*B
NLK: Nemo-like kinase
PBEC: Primary bronchial epithelial cell
PBMC: Peripheral blood mononuclear cell
PF: Pulmonary fibrosis
PKC: Protein kinase C
PSMCs: Parabronchial smooth muscle cells
PTX: Pentoxifylline
Ror: Receptor tyrosine kinase-like orphan receptor
RYK: Receptor-like tyrosine kinase
SD: Sprague Dawley
SFRP1: Secreted frizzled-related protein-1
shRNA: Short hairpin RNA
siRNA: Small interfering RNA
SMA: Smooth muscle actin
SPA: Surfactant protein A
SPD: Surfactant protein D
TAK1: Transforming growth factor-*β* activated kinase-1
TCF/LEF1: T-cell factor/lymphoid enhancer factor 1
TGF-*β*: Transforming growth factor-beta
Th2: Helper 2 T-cells
TNF*α*: Tumor necrosis factor *α*
UIP: Usual interstitial pneumonia
WIF1: Wnt inhibitory factor 1
WISP1: Wnt1 inducible signaling pathway protein-1
Wnt: Wingless-type MMTV integration site.
